# The Association between Adiponectin Single Nucleotide Polymorphisms and Side Effects of Isotretinoin in Acne Patients

**DOI:** 10.1155/2020/3176521

**Published:** 2020-04-29

**Authors:** Munir Garba, Omar F. Khabour, Karem H. Alzoubi, Ahmed Abu-Siniyeh, Firas Al-Qarqaz

**Affiliations:** ^1^Department of Medical Laboratory Sciences, Faculty of Applied Medical Sciences, Jordan University of Science and Technology, Irbid, Jordan; ^2^Department of Clinical Pharmacy, Jordan University of Science and Technology, Irbid, Jordan; ^3^Clinical Laboratory Sciences Department, College of Applied Medical Sciences, Taif University, Taif, Saudi Arabia; ^4^Department of Dermatology, Faculty of Medicine, Jordan University of Science, Technology, Irbid, Jordan

## Abstract

**Background:**

Acne is a common condition of pilosebaceous follicle especially among young. Clinically, the most used medication in the treatment of moderate to severe acne is oral isotretinoin. However, interindividual variability in therapeutic response to isotretinoin and many side effects such as musculoskeletal pain, headache, and alteration in lipid profile can be seen with this treatment.

**Aim:**

In this study, the effect of genetic polymorphisms, rs2241766 and rs1501299, of the adiponectin gene was investigated in relation to the side effects of isotretinoin-treated young adult acne patients (*n* = 230).

**Methods:**

Several biochemical parameters were measured at baseline and after treatments with isotretinoin. The ADIPOQ gene SNPs, rs2241766 and rs1501299, were genotyped in 230 patients.

**Results:**

Alterations in lipid profile with a significant increase of ALT (*P*=0.007) were detected after isotretinoin treatment. Moreover, percentage change in HDL following isotretinoin treatment was significantly associated with rs1501299 (*P*=0.008). On the other hand, no associations between examined SNPs and side effects of isotretinoin and other lipid parameters (total cholesterol, LDL, and triglycerides) or liver function enzymes (ALT and AST) were detected.

**Conclusions:**

Current findings showed that rs1501299 of the ADIPOQ gene might be associated with changes in HDL level in acne patients following treatment with isotretinoin.

## 1. Introduction

Acne is a common skin condition of the pilosebaceous follicle, identified by a variety of noninflamed (comedons) and inflammatory lesions [1, 2], which primarily affects the face, upper trunk, upper arms, and back [3]. The prevalence and the severity of acne is highest among the adolescence, affecting up to 91% of males and 79 % of females of this group [4] and often persist well in adulthood [5].

The treatment of acne is based on the severity and the appearance of acne lesions [6]. Oral isotretinoin (13-*cis*-retinoic acid) is considered to be the most effective medication in the treatment of moderate-to-severe form of acne vulgaris [7]. However, the use of isotretinoin is associated with serious side effects [8], including depression and suicidal behavior [9, 10], elevated liver enzymes, teratogenicity, arthralgia, headache, and myalgia [11], and acute kidney injury [12, 13].

Adiponectin, a 30 KDa product of the ADIPOQ gene [14], is an abundant protein in human adipose tissues [15]. In addition, the circulating level of adiponectin in blood ranges between 2 and 30 mg/L in human [16] and about 1.5 times higher in females than males [17]. Adiponectin plays an important role in body metabolism and immune response [18, 19]. Changes in adipocytokine levels have been reported to be associated with isotretinoin medication [20–24]. For example, isotretinoin treatment of acne patients causes a significant increase in adiponectin plasma level [21, 22] and significant decrease in leptin level [22]. The ADIPOQ gene contains a number of SNPs, mostly of unknown functions. Among such SNPs are rs1501299 and rs2241766 that have been shown to be of clinical significance [25–28]. This includes modulation of disease susceptibility and the clinical outcomes to therapeutic agents [15, 26, 29, 30]. In this study, we aimed to investigate the relationship between genetic polymorphisms in the adiponectin gene, rs1501299 and rs2241766, and the side effects response in acne patients treated with 13-*cis*-retinoic acid among Jordanian population.

## 2. Materials and Methods

### 2.1. Study Design and Study Subjects

This cross-sectional correlation study was based on Jordanian population, in which 230 patients suffering from acne were recruited to participate in the study. Patients were enrolled in the study through collaboration with the Dermatology Department of JUST-Medical Center (Irbid), King Abdullah University Teaching Hospital (KAUH, Irbid), and Prince Hamza Hospital (Amman). All patients had established acne disease and were diagnosed on the basis of global assessments of acne. The inclusion criteria used were patients with diagnosis of acne planned to start treatment by oral isotretinoin (40 mg/day) and age 14–65 years with available information about baseline and after 1 month treatment lipid profiles and liver function tests.

The exclusion criteria were patients with habitual excessive use of alcohol, elevated lipid profile and liver enzymes (serum ALT or AST) prior to study, chronic medical illness (other than acne), and patients with concomitant and severe health problems and also any patient who refused to give informed consent. The Institutional Review Board (IRB) of Jordan University of Science and Technology approved the study.

### 2.2. Sample Collection and Analysis

Fasting blood sample was taken prior to the first course of isotretinoin from each subject. Initially, 3 ml of blood sample was transferred to an EDTA tube and stored at –20°C for genetic analysis. The remaining 2 ml was transferred to plain tube, and serum was obtained and stored at −20°C prior to analysis of biochemical markers. The serum levels of total cholesterol (TChol), LDL, HDL, triglyceride (TG), aspartate aminotransferase (AST), and alanine aminotransferase (ALT) were measured in the diagnostic laboratories of KAUH using a biochemistry analyzer (Roche Diagnostics, Mannheim, Germany). The procedure was repeated from each patient a month after treatments with 13-*cis*-retinoic acid.

Patients enrolled in the study were interviewed using a structured questionnaire. Patient variables and data necessary for further analysis (the major demographic variables including age, gender, and education status) were recorded. Height, weight, and clinical history and current medication other than isotretinoin were obtained from the patient's medical files. Common adverse effect profiles such as muscle pain, joint pain, headache, and nose bleeds, after oral isotretinoin therapy, as reported by the patients were recorded.

The BMI was calculated using the following equation: BMI = weight (kg)/height (m^2^), and patients were categorized as underweight (<18 Kgm^**−**2^), normal weight (14–24.9 Kgm^−2^), overweight (25–29.9 Kgm^−2^), and obese (>30 Kgm^−2^).

Assessment of depression symptoms was performed based on Arabic translated version of Beck Depression Inventory. Based on the reported symptoms, the patients were initially classified into two groups: group 1 comprised patients who did not express depression symptoms after one month of isotretinoin therapy and group 2 comprised patients with depression symptoms after isotretinoin therapy.

### 2.3. Genotyping of ADIPOQ Gene

The rs1501299 and rs2241766 SNPs in the ADIPOQ gene were selected based on their clinical significance on body metabolism. Genomic DNA was extracted from blood samples using the Quick-gDNA^TM^ MicroPrep mini kit (Zymo Research Corp, California USA), according to the manufactures protocol. PCR reaction was used to amplify the desired regions of the ADIPOQ gene at rs1501299 and rs2241766 [31, 32]. The PCR reaction was carried out using a Bio-Rad thermocycler (Bio-Rad Company) using the following protocols: precycling denaturation performed at 94°C for 7 minutes, 40 cycles denaturation for 35 seconds at 94°C, annealing for 35 seconds at 58°C, extension at 72°C for 35 seconds, and final extension for 72°C at 7 minutes. The primer sequences used for the amplification of both ADIPOQ gene SNPs, rs1501299 (F:5′GTC TAG GCC TTA GTT AAT AAT GAA TG-3′, R:5′GAG AAA GGA GAT CCA GGT AAG A-3′) and rs2241766 (F:5′-CTG AGA TGG ACG GAG TCC TTT-3′, R:5′-CCA AAT CAC TTC AGG TTG CTT-3′). After PCR, sample amplification was confirmed by gel electrophoresis, followed by detection using gene snap image accusation software (Syngene, Synoptics Ltd).

PCR-RFLP was performed on the representative of each sample. For PCR-RFLP, 10 *µ*l of each amplified PCR product was subjected to *SmaI* (New England Bio Lab (UK) Ltd) restriction endonucleases digestion at 25°C incubation followed by heat inactivation at 20°C after complete digestion, and *BsmI* (New England Bio Lab (UK) Ltd) restriction endonucleases digestion, which recognizes the G/T allele at 65°C incubation followed by heat inactivation at 85°C. The products of digestion were resolved by electrophoresis (Bio-Rad power gel) in 3% agarose gel, stained with ethidium bromide. Gel photography was carried out using gene snap image accusation software (Syngene, Synoptics Ltd).

### 2.4. Statistical Analysis

Statistical analysis was carried out using SPSS software (SPSS, Chicago, IL, USA). Results were presented as mean (±SD). Comparisons were achieved using the chi-squared test, Student's *t*-test, or ANOVA as appropriate. A corrected *P* value (<0.0084) for multiple comparisons (6 variables) was considered statistically significant.

## 3. Results

Initially, 230 patient's blood samples and data were obtained, but due to the incomplete information, 9 patients were excluded in the final analysis. Therefore, only 221 patients were included in this study. The demographic characteristics of the patients are presented in Table 1. Of these, 94.7% were females with female to male ratio being 9 : 1. The mean age calculated was 23.9 years old (SD, 6.498), and majority of patients were in between 21 and 30 years old. The mean BMI was 24.34 and about 90% of the patients were single and nonsmokers.

Apart from skin dryness, which was seen in all patients (a treatment effect), the most common adverse effects associated with isotretinoin treatment in the studied population were joint pain, headache, and mucosal dryness/nose bleeds. Moreover, serum levels of LDL, TChol, TG, AST, and ALT were significantly elevated one month after oral isotretinoin treatment, while serum HDL was decreased (Table 2).

The distribution of the genotypic and allelic frequencies of rs2241766 was as follows: 171 (74.35%) patients were homozygote for the wild-type TT alleles, 57 (24.78%) were heterozygote for TG alleles, and 2 (0.87%) were homozygote for GG mutant alleles. The frequency of the T allele was 399 (86.74%) while for the G allele was 61 (13.2%). This signified that the T alleles occurred more frequently than G alleles in the population studied with respect to rs2241766 of ADIPOQ.

To investigate whether rs2241766 SNPs of the ADIPOQ genotype had any effects on the alteration of lipid profile and liver enzymes after administration of isotretinoin, genotypes of rs2241766 TT and TG/GG were analyzed with respect to HDL, LDL, TG, TChol, and liver enzymes (Table 3). No significant association was found with rs2241766 genotypes and alteration of biochemical parameters, suggesting that rs2241766 might not be a contributing factor in alteration of biochemical parameters after the use of isotretinoin in acne patients.

The genotypic and allelic frequencies of rs1501299 were distributed as follows: 118 (51.3%) were homozygote for the wild-type allele GG, 112 (48.7) patients were heterozygote for GT, and none of the patients were homozygote for the mutant allele TT of rs1501299. The frequency of the G allele was 348 (75.7%) while for the T allele was (112) 24.3%.

To evaluate whether the rs1501299 genotype had any effect on alteration of lipid profile and liver enzymes, HDL, LDL, TG, TChol, AST, and ALT were analyzed with respect to the rs1501299 genotypes of acne patients Table 4. No significant association was observed between alteration of lipid profile and liver enzymes after treatments with oral isotretinoin. But, percent change by treatment of HDL showed an association with the GG and GT genotypes of rs1501299.

Each genotype was tested according to the side effect to investigate whether genetic influence of rs2241766 and rs1501299 could possibly be associated with the most common side effects of isotretinoin treatment. Neither rs2241766 nor rs1501299 polymorphism was found to be associated with the side effect of isotretinoin as shown in Table 5.

To test whether both the polymorphisms of adiponectin gene, rs2241766 and rs1501299 genotypes, are associated with depression symptoms of isotretinoin, depression status was compared among genotypes in rs2241766 TT and TG/GG (*Pp* value = 0.066) and in rs1501299 GG and GT (*P* value = 0.052). Therefore, neither rs2241766 nor rs1501299 is statistically associated with depression symptoms of isotretinoin.

## 4. Discussion

The aims of this study were to determine the most common side effects of isotretinoin treatment among Jordanian acne patients and to investigate the correlation between adiponectin gene variants, rs1501299 and rs2241766, and severity of acne and side effects of isotretinoin. The side effects of oral isotretinoin treatment are similar to some extent with that reported in other populations. In addition, the rs1501299 and rs2241766 SNPs seem to play a limited effect on side effects of isotretinoin-treated acne patients.

Isotretinoin remains the single and most important therapeutic agent that impacts virtually in all of the major etiological factors that contribute to the formation of acne [7]. The drug is capable of inducing remission after an adequate course of therapy by influencing cellular division/differentiation and significant suppression in the sebum production [33].

Regarding the adverse effect of isotretinoin on lipid and liver enzyme levels, our results were consistent with previous studies from different populations. The drug increased the cholesterol, triglyceride, and liver enzymes at a high level despite of its respectable reputation in curing acne [34−36]. On the other hand, it has been demonstrated that these high levels of lipids and liver enzymes returned normal after isotretinoin was stopped. In other retrospective study, lipids and liver enzymes were tested after 3 and 6 months following oral isotretinoin administration. Significant increases in TG, LDL, and AST levels were recorded during this period, while HDL levels were decreased. In this cohort of patients, lipids were more affected with isotretinoin than liver enzymes, and this confirms the need for regular assessment of LFTs and lipid profiles during treatment with isotretinoin as suggested by the treatment guidelines [34, 35]. These recommendations were affirmed with a later study as the results were consistent with what has been found previously, that is, variable increase in several lipid profiles when a low-dose isotretinoin was administrated by the patients. This work also emphasized on continuous monitoring of lipid profile, especially in patient with risk factors of metabolic syndrome [37]. A recent study showed that a slight-to-modest increase of lipid and liver enzyme levels always occurs when isotretinoin is taken. They came to a conclusion that frequent follow-up is not needed because the adverse effect of isotretinoin is revocable [38].

The most common reported side effects in this group of patients were mucocutaneous (dryness/nose bleeds), musculoskeletal (joint pain), and neurological (headaches). There was no statistically significant correlation with ADIPOQ gene carries of rs1501299 and rs2241766. A previous report from Turkey on 19 acne patients with 0.5 mg/kg/day convectional therapy of isotretinoin showed that the most common side effects reported were dry, chapped lips by 100 %, dry skin by 78%, nose bleeds in 11%, and fatigue in 5% of patients. None of the patients in this group reported either joint pains or muscle pains [39]. Attempt was made to correlate ADIPOQ gene carries of rs1501299 and rs2241766 and common side effects of 13-*cis*-retinoic acid treatment; however, the result did not reach any statistical significance.

The results showed that treatment with oral isotretinoin resulted in alteration in lipid profile and ALT and AST liver enzymes, with the marked decrease of HDL. This study found an association between the % change of HDL by treatment and GT of rs1501299, but no significant association was found between alteration in lipid profile and liver enzymes with GT and TG/GG polymorphisms of rs1501299 and rs2241766, respectively. The association between the % change of HDL and rs1501299 SNP highlights the importance of giving consideration to pharmacogenomics in oral isotretinoin treatments. The rs1501299 SNP has been shown to be associated with lipid parameters and arterial stiffness in many populations [41−44].

The present study showed that the frequency of the rs2241766 G and rs1501299 T minor alleles was 13% and 24%, respectively. The frequency of the rs2241766 G allele was similar to that of Iraqi (12%) population [40] and slightly lower than that of Egyptian (25.7%), Saudi (28.5%), and Tunisian (19%) populations [25, 41, 43]. With respect to the rs1501299 T allele, the observed frequency was higher than that reported among Saudi (1.6–4.0%) population [25] and slightly lower than that reported among Tunisian (32%) and Egyptian (31%) populations [41, 43]. A previous study showed that the rs2241766 and rs1501299 SNPs were in weak linkage disequilibrium (*r*^2^ = 0.063, D' = 0.621) [42]. However, linkage disequilibrium between these SNPs was not tested in the current investigation.

Among the limitations of the study is the relatively small sample size. In addition, genetic effects are normally affected by environmental factors. In the current study, the observed impact of rs1501299 on HDL percent change by isotretinoin treatment might be affected by diet factors. Therefore, future studies with a larger sample and controlling for diet factors are needed to confirm the present findings. Examining the impact of genetic variation on side effects of isotretinoin at the end of the treatment is strongly recommended in future studies.

In conclusion, the examined rs1501299 and rs2241766 SNPs seem to play a limited effect on the side effects of isotretinoin among acne patients. The results need to be confirmed in other populations.

## Figures and Tables

**Table 1:** Demographics of patients participated in the study (*n* = 221).

Variable	Patients, *N* (%)
Age	
Mean (SD)	23.9 (6.498)

Age groups (years)	
14–20	69 (30.4)
21–30	122 (53.0)
31–40	25 (10.9)
>40	4 (1.70)

Gender	
Male	36 (5.3%)
Female	185 (94.7%)

Marital status	
Single	184 (72.8%)
Married	37 (27.2%)

Smoking	
Yes	15 (6.8%)
No	206 (93.2%)

BMI	24.34 (3.52)

Common side effects	
Joint pain	167 (72.3%)
Nose bleeds	122 (52.8%)
Headache	124 (53.7%)

**Table 2:** Alterations in lipid profile and liver transaminases after use of oral isotretinoin.

Parameter	Reference value^*∗*^	Baseline, mean (SEM)	After treatment, mean (SEM)	*P* value
HDL (mmol/L)	0.9–1.5	1.44 (0.02)	1.31 (0.02)	≤0.001
LDL (mmol/L)	2.6–4.8	2.35 (0.04)	2.82 (0.05)	≤0.001
TChol (mmol/L)	Up to 5.2	4.17 (0.06)	4.68 (0.06)	≤0.001
Triglycerides (mmol/L)	Up to 4.8	0.99 (0.04)	1.30 (0.05)	≤0.001
ALT (U/L)	Up to 41	15.91 (0.54)	17.43 (0.68)	0.007
AST (U/L)	Up to 35	19.01 (0.40)	21.47 (0.51)	≤0.001

Data were obtained from the paired *t*-test. Correlation is significant at *Pp* value less than 0.05. SEM: standard error of the mean. HDL: high-density lipoprotein. LDL: low-density lipoprotein. TChol: total cholesterol. AST: aspartate aminotransferase. ALT: alanine aminotransferase. ^*∗*^Laboratory normal reference value of lipid profile and liver enzymes. Source: Biochemistry Laboratory of Jordan University of Science and Technology, Health Center, Irbid.

**Table 3:** Means and standard deviations of biochemical parameters (baseline, after treatment, and percentage of change by treatment) for the genotype of rs2241766.

Parameter	Genotypes		*P* value
	TT, mean (SD)	TG/GG, mean (SD)	
HDL (mmol/L)			
Baseline	1.44 (0.377)	1.42 (0.315)	0.930
After treatment	1.304 (0.310)	1.34 (0.327)	0.553
% change by treatment	−7.4% (14.86)	−4.2% (19.41)	0.129

LDL (mmol/L)			
Baseline	2.35 (0.70)	2.32 (0.67)	0.275
After treatment	2.85 (0.75)	2.74 (0.83)	0.570
% change by treatment	2.5% (25.7)	1.9% (20.9)	0.699

TChol (mmol/L)			
Baseline	4.22 (0.85)	4.03 (1.08)	0.636
After treatment	4.71 (0.91)	4.57 (1.10)	0.339
% change by treatment	12.8% (14.4)	1.6% (20.4)	0.074

TG (mmol/L)			
Baseline	0.99 (0.57)	1.00 (0.66)	0.642
After treatment	1.31 (0.71)	1.27 (0.71)	0.872
% change by treatment	43.3% (58.76)	42.3% (78.0)	0.219

AST (U/I)			
Baseline	19.5 (6.60)	17.59 (4.00)	0.118
After treatment	21.84 (7.64)	20.39 (7.77)	0.337
% change by treatment	15.3% (35.0)	16.5% (30.7)	0.664

ALT (U/I)			
Baseline	16.25 (8.34)	14.97 (7.63)	0.338
After treatment	17.17 (8.26)	18.20 (14.73)	0.142
% change by treatment	13.2% (46.04)	21.5% (51.88)	0.396

Student's *t*-test was used. *P* value is significant at less than 0.05. HDL: high-density lipoprotein. LDL: low-density lipoprotein. TChol: total cholesterol. AST: aspartate aminotransferase. ALT: alanine aminotransferase.

**Table 4:** Means and standard deviations of biochemical parameters (baseline, after treatment, and percentage of change by treatment) for the genotype of rs1501299.

Parameter	Genotypes		*P* value
	GG, mean (SD)	GT, mean (SD)	
HDL (mmol/L)			
Baseline	1.42 (0.366)	1.453 (0.358)	0.690
After treatment	1.29 (0.305)	1.35 (0.322)	0.574
% change by treatment	−8.2% (13.5)	−5.6% (18.6)	0.0076

LDL (mmol/L)			
Baseline	2.33 (0.598)	2.36 (0.779)	0.082
After treatment	2.80 (0.684)	2.85 (0.826)	0.217
% change by treatment	22.3% (23.5)	24.2% (25.8)	0.709

TChol (mmol/L)			
Baseline	4.11 (0.868)	4.24 (0.959)	0.333
After treatment	4.60 (0.874)	4.75 (1.045)	0.216
% change by treatment	13.8% (17.1)	13.3% (15.2)	0.869

TG (mmol/L)			
Baseline	0.99 (0.56)	0.99 (0.63)	0.395
After treatment	1.25 (0.68)	1.36 (0.74)	0.572
% change by treatment	36.7% (64.7)	49.7% (62.9)	0.767

AST (U/I)			
Baseline	19.15 (5.26)	18.85 (6.88)	0.456
After treatment	21.54 (6.99)	21.39 (8.39)	0.618
% change by treatment	15.3% (36.2)	15.9% (31.4)	0.808

ALT (U/I)			
Baseline	15.56 (6.20)	16.30 (9.85)	0.093
After treatment	16.73 (7.69)	18.17 (12.45)	0.268
% change by treatment	12.8% (47.6)	18.0% (47.6)	0.819

^∗^Student's *t*-test was used. *P* value is significant at less than 0.05. HDL: high-density lipoprotein. LDL: low-density lipoprotein. TChol: total cholesterol. AST: aspartate aminotransferase. ALT: alanine aminotransferase.

**Table 5:** Genotypes frequencies of tested polymorphisms according to common adverse effects of isotretinoin treatment.

Parameter		rs2241766			*P* value	rs1501299		*P* value
		GG, number (%)	TG, number (%)	TT, number (%)		GG, number (%)	GT, number (%)	
Joint pain	Yes	2 (0.9)	43 (19.5)	122 (55.5)	0.708	87 (39.5)	80 (36.4)	0.926
	No	0 (0.0)	13 (5.9)	40 (18.2)		28 (12.7)	25 (11.4)	

Mucosal dryness/nose bleed	Yes	1 (0.5)	32 (14.5)	89 (40.5)	0.948	65 (29.5)	57 (25.9)	0.739
	No	1 (0.5)	24 (10.9)	73 (33.2)		50 (22.7)	48 (21.8)	

Headache	Yes	0 (0.0)	33 (15.0)	91 (41.4)	0.255	70 (31.8)	54 (24.5)	0.158
	No	2 (100)	23 (41.1)	71 (43.8)		45 (20.5)	51 (23.2)	

## Data Availability

The data used to support the findings of this study are available from the corresponding author upon request.
